# Full-Endoscopic Unilateral Laminotomy for Bilateral Decompression Under Surgeon-Directed Local Anesthesia in Older Patients with Lumbar Spinal Stenosis

**DOI:** 10.3390/medicina62091761

**Published:** 2026-09-12

**Authors:** Yong Ahn, Sungsoo Bae, Jae-Ouk Lee

**Affiliations:** Department of Neurosurgery, Kyung Hee University Hospital at Gangdong, Kyung Hee University College of Medicine, Seoul 05278, Republic of Korea; sungsoobae09@gmail.com (S.B.); leejaeouk@naver.com (J.-O.L.)

**Keywords:** lumbar spinal stenosis, endoscopic spine surgery, unilateral laminotomy for bilateral decompression, local anesthesia, older patients

## Abstract

*Background and Objectives*: Older patients with lumbar spinal stenosis often have comorbidities and limited physiological reserve that complicate conventional decompression under general anesthesia. This study aimed to evaluate the surgical technique and preliminary 1-year clinical outcomes of full-endoscopic unilateral laminotomy for bilateral decompression (FE-ULBD) performed under surgeon-directed local anesthesia. *Materials and Methods*: This retrospective cohort study included 24 consecutive patients aged ≥ 75 years who underwent FE-ULBD under surgeon-directed local anesthesia for symptomatic central lumbar spinal stenosis between March 2024 and April 2025. Clinical outcomes were evaluated using the visual analog scale (VAS) for leg pain, Oswestry Disability Index (ODI), and modified MacNab criteria. Additionally, perioperative outcomes and complications were analyzed. *Results*: The mean age of patients was 80.5 ± 3.8 years. The mean VAS scores improved from 8.46 ± 0.72 preoperatively to 2.08 ± 0.83 at 12 months, while ODI scores improved from 68.83% ± 8.07% to 18.34% ± 13.74% (both *p* < 0.001). Of the 24 patients, 21 (87.5%) achieved excellent or good outcomes. Procedure-related complications included an incidental dural tear in one patient (4.2%) and transient postoperative dysesthesia in 4 patients (16.7%). No major anesthesia-related complications or revision surgeries were observed. *Conclusions*: FE-ULBD under surgeon-directed local anesthesia was feasible and associated with improvements in pain and function at 12 months in carefully selected older patients. These preliminary findings warrant confirmation in larger prospective studies with a comparator cohort of older patients receiving general anesthesia.

## 1. Introduction

Lumbar spinal stenosis is a common degenerative spinal disorder in older adults and a major cause of neurogenic claudication, radicular leg pain, and functional limitation. As populations continue to age, an increasing number of older patients require surgical treatment after failed conservative management. Previous randomized and long-term studies have demonstrated that decompressive surgery provides superior symptom relief and functional improvement compared with continued nonoperative treatment in appropriately selected patients with symptomatic lumbar spinal stenosis [1,2].

Despite these established benefits, surgical decision-making in older patients remains challenging. Older patients frequently present with multiple medical comorbidities, reduced physiological reserve, and increased susceptibility to perioperative complications. Although conventional decompression under general anesthesia is effective, the physiological stress associated with general anesthesia and extensive surgical exposure may delay postoperative recovery and increase systemic complications in medically vulnerable older adults [3,4,5]. Accordingly, increasing attention has been directed toward treatment strategies that reduce surgical invasiveness and anesthesia-related burden while maintaining effective neural decompression.

Unilateral laminotomy for bilateral decompression (ULBD) is a well-established motion-preserving technique for central lumbar spinal stenosis. By achieving bilateral neural decompression through a unilateral approach, ULBD minimizes tissue disruption while preserving the posterior stabilizing structures [6]. Recent advances in endoscopic visualization, irrigation systems, high-speed burrs, and dedicated instruments have further expanded the role of full-endoscopic spine surgery in the treatment of degenerative lumbar spinal stenosis [7,8,9,10,11]. Full-endoscopic ULBD (FE-ULBD) enables precise neural decompression through a small working channel while minimizing muscle injury and blood loss, thereby facilitating rapid postoperative recovery [10,11,12,13,14]. These characteristics make FE-ULBD particularly attractive for older patients, in whom minimizing surgical trauma is an important therapeutic objective.

Along with these technical advances, we adopted a surgeon-directed local anesthesia protocol for FE-ULBD that integrates stepwise local anesthetic infiltration, conscious sedation, monitored anesthesia care (MAC), and endoscopically guided epidural anesthesia. This protocol was designed to facilitate full-endoscopic decompression while preserving continuous patient communication and real-time neurological feedback throughout the procedure. Previous studies have demonstrated favorable clinical outcomes of endoscopic decompression performed under local anesthesia, particularly in medically vulnerable older patients with foraminal or lateral recess stenosis [15,16,17,18].

However, evidence regarding the application and technical aspects of FE-ULBD performed under surgeon-directed local anesthesia remains limited. Therefore, the present study aimed to describe the surgical technique of FE-ULBD performed under surgeon-directed local anesthesia, which incorporates stepwise local anesthesia and endoscopically guided epidural anesthesia, and to evaluate its 1-year clinical outcomes and safety in patients aged 75 years or older with symptomatic central lumbar spinal stenosis.

## 2. Materials and Methods

### 2.1. Patient Population

This retrospective cohort study was based on a prospectively maintained spine surgery registry. Between March 2024 and April 2025, patients aged 75 years or older who underwent FE-ULBD under surgeon-directed local anesthesia for central lumbar spinal stenosis were screened. Only patients with a minimum clinical follow-up of 12 months were included in the final analysis. This study was approved by the Institutional Review Board of Kyung Hee University Hospital at Gangdong (KHNMC 2026-04-033). The requirement for informed consent was waived due to the retrospective nature of the study.

Inclusion criteria were: (1) age ≥ 75 years; (2) neurogenic intermittent claudication and/or bilateral lower-extremity radicular symptoms attributable to lumbar spinal stenosis; (3) single-level central canal stenosis confirmed by magnetic resonance imaging (MRI) and computed tomography (CT); (4) severe central stenosis classified as Schizas grade C or D on preoperative MRI [19]; and (5) persistent symptoms despite at least 3 months of conservative treatment, including medication, physical therapy, and epidural injections. Exclusion criteria were: (1) dynamic segmental instability on flexion–extension radiographs; (2) grade II or higher degenerative spondylolisthesis; (3) isolated lumbar disc herniation without central stenosis; (4) predominant foraminal or extraforaminal stenosis requiring an alternative decompression strategy; (5) predominant mechanical low-back pain without radicular symptoms; and (6) spinal infection, tumor, trauma, inflammatory disease, or other nondegenerative spinal disorders.

The preoperative comorbidity burden was assessed through a retrospective review of medical records. The American Society of Anesthesiologists (ASA) physical status was determined based on documented preoperative systemic diseases and functional status according to the ASA criteria [20]. The age-unadjusted Charlson Comorbidity Index (CCI) was calculated using the original weighted comorbidity categories, and the age-adjusted CCI was calculated separately by adding 3 points for age 70–79 years and 4 points for age ≥ 80 years [21]. Hierarchical CCI conditions were counted using the highest applicable weight without duplication. Hypertension, coronary artery disease, Parkinson’s disease, depression, benign prostatic hyperplasia, and dyslipidemia were tabulated separately when present because they are not independently weighted in the CCI.

### 2.2. Surgical Technique

**Figure 1:**
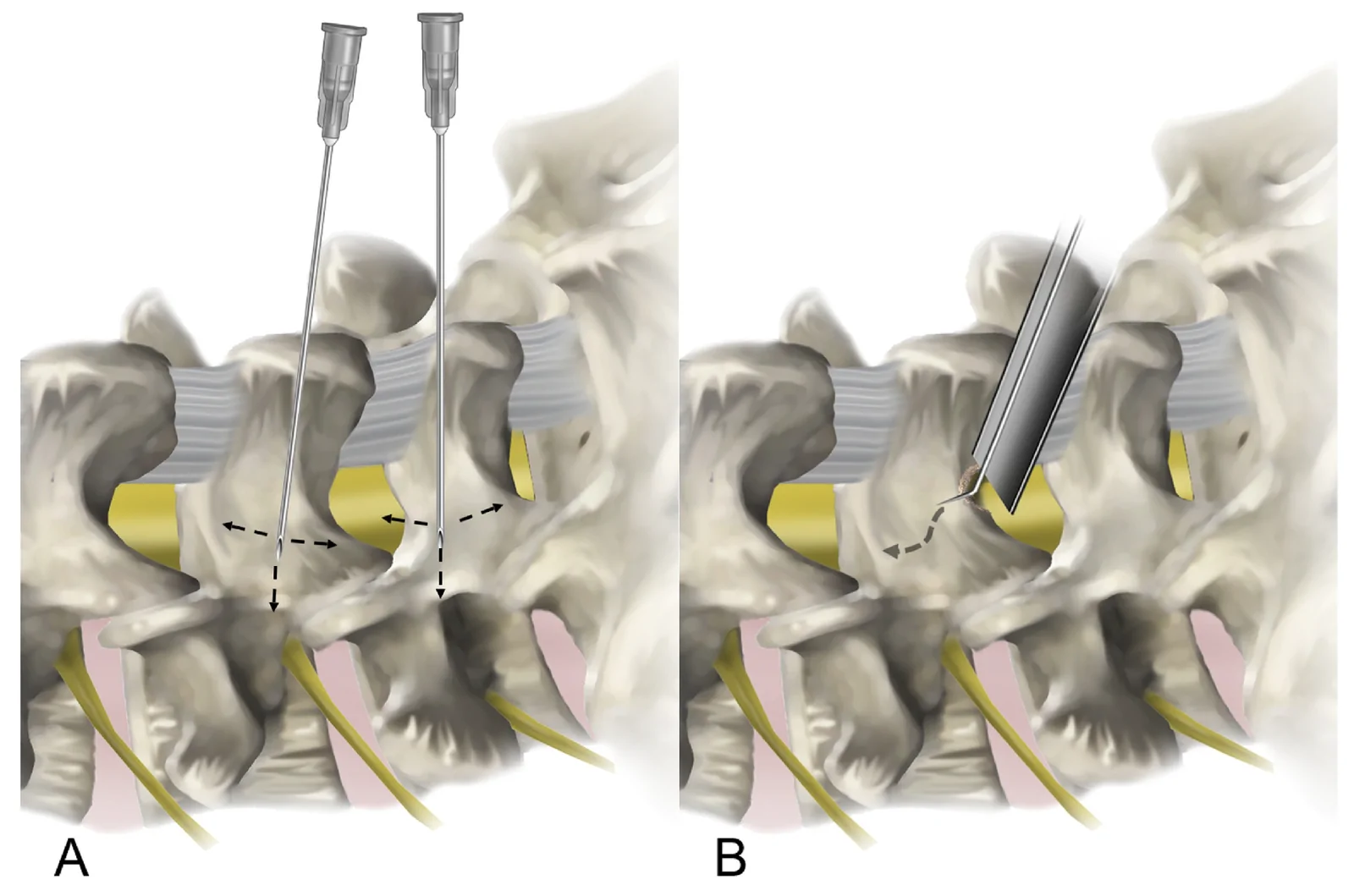
Stepwise local anesthesia protocol for FE-ULBD under surgeon-directed local anesthesia. (**A**) Stepwise infiltration of local anesthetic into the skin, paraspinal muscles, and periosteum before endoscopic access. The dashed arrows indicate the spread of the local anesthetic through the surrounding soft tissues. (**B**) After creation of a small midline laminotomy window, a curved-tip spinal needle is advanced into the epidural space under direct endoscopic visualization. Approximately 10 mL of 0.75% ropivacaine is slowly injected, followed by a waiting period of approximately 2 min to achieve adequate epidural anesthesia before neural decompression. The dashed arrow indicates the distribution of the epidurally injected local anesthetic within the epidural space.

**Figure 2:**
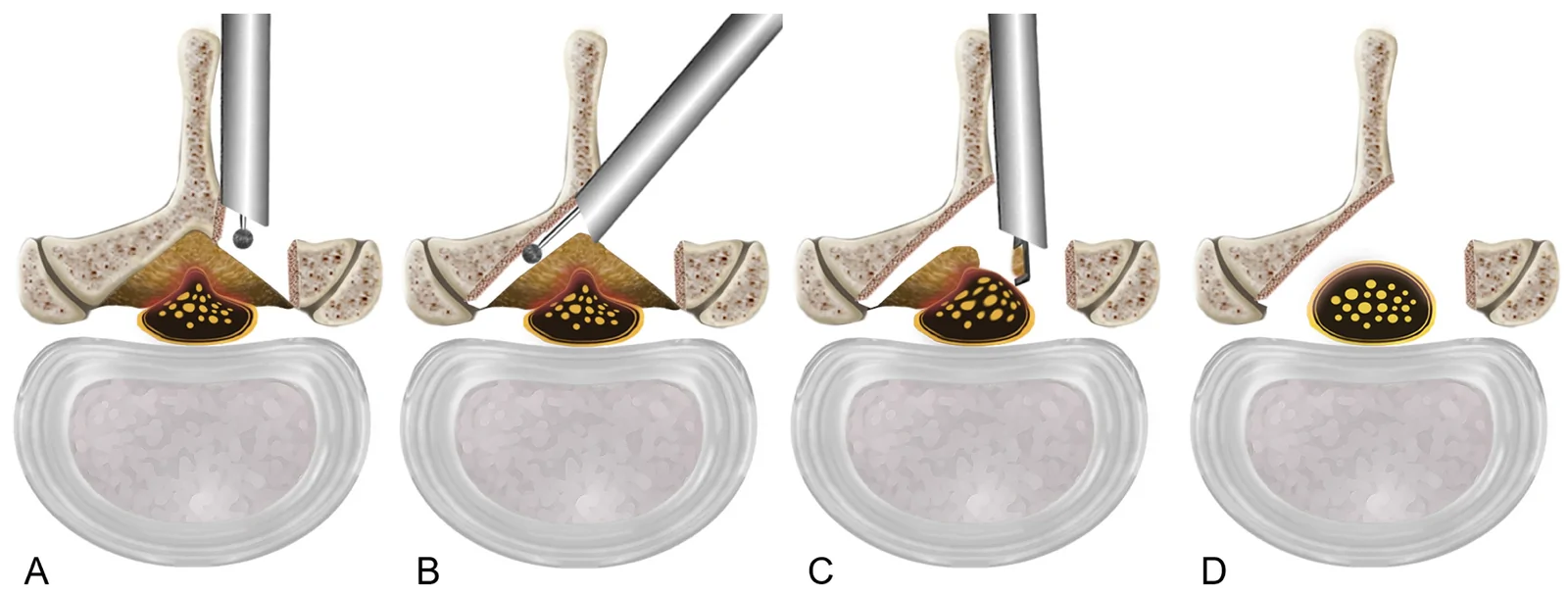
Schematic illustrations of FE-ULBD. (**A**) Ipsilateral laminotomy is initiated through the interlaminar approach using a high-speed burr while preserving the ligamentum flavum. (**B**) Bilateral decompression is extended to the contralateral side using an over-the-top approach with progressive undercutting of the contralateral lamina. (**C**) After adequate bony decompression, the hypertrophic ligamentum flavum is carefully detached and removed under endoscopic visualization. (**D**) Final appearance after bilateral neural decompression demonstrating complete expansion of the dural sac and decompression of both lateral recesses.

**Figure 3:**
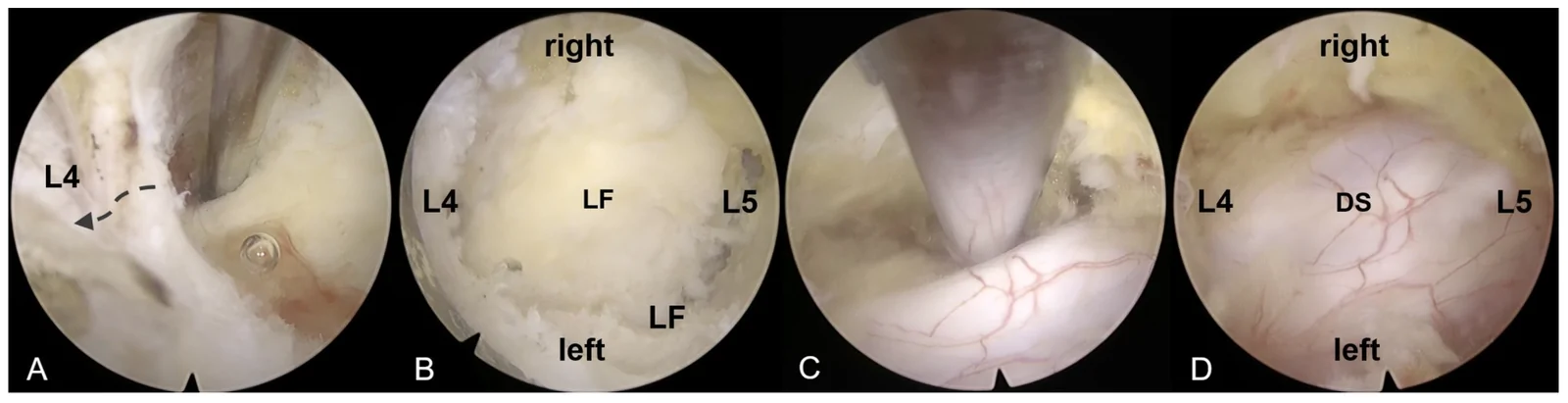
Intraoperative endoscopic findings during FE-ULBD. (**A**) Endoscopically guided epidural anesthesia through a small laminotomy window. A curved-tip spinal needle is introduced into the epidural space under direct endoscopic visualization. The dashed arrow indicates the spread of the epidurally injected local anesthetic. (**B**) Over-the-top contralateral decompression following ipsilateral laminotomy. The hypertrophic ligamentum flavum (LF) is intentionally preserved as a protective layer, whereas its bony attachment has been detached following bilateral undercutting of the laminae. (**C**) After removal of the hypertrophic LF, the laminotomy margin is refined using an endoscopic punch to complete canal decompression. (**D**) Final endoscopic view demonstrating complete bilateral neural decompression with full expansion of the dural sac (DS). L4, fourth lumbar vertebra; L5, fifth lumbar vertebra; LF, ligamentum flavum; DS, dural sac.

The surgical procedure consisted of three sequential steps: (1) preoperative planning based on the patient’s symptoms and radiologic findings, including MRI and CT, to determine the target level and extent of decompression; (2) surgeon-directed local anesthesia with full-endoscopic interlaminar access and endoscopically guided epidural anesthesia (Figure 1); and (3) ULBD under full-endoscopic visualization (Figure 2 and Figure 3).

#### 2.2.1. Preoperative Planning

**Figure 4:**
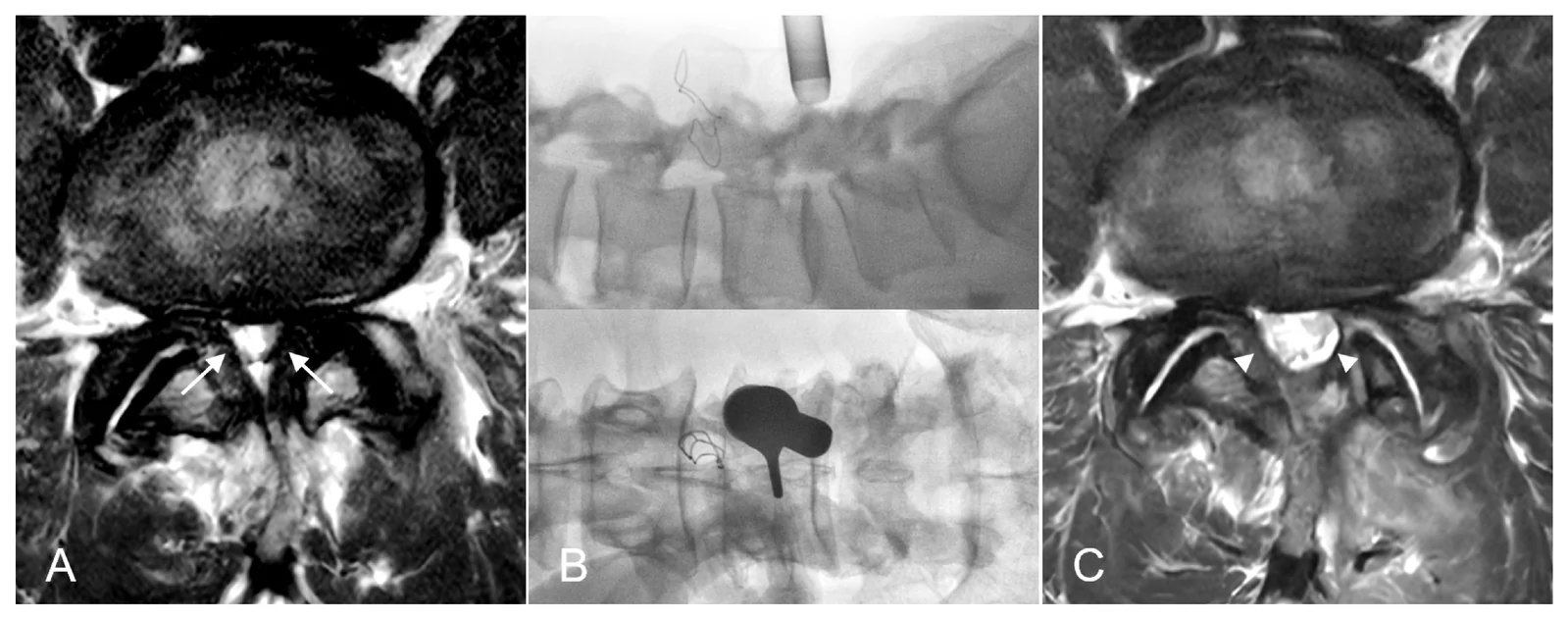
Representative case of a 78-year-old man with neurogenic intermittent claudication and bilateral radicular pain who underwent FE-ULBD under surgeon-directed local anesthesia. (**A**) Preoperative axial T2-weighted MRI demonstrating severe central lumbar spinal stenosis at the L4–5 level caused by hypertrophic ligamentum flavum and facet hypertrophy (arrows). (**B**) Intraoperative lateral (upper) and anteroposterior (lower) fluoroscopic images showing correct placement of the endoscopic working sheath at the L4–5 interlaminar space during FE-ULBD. (**C**) Postoperative axial T2-weighted MRI demonstrating successful over-the-top bilateral decompression with expansion of the dural sac and bilateral lateral recesses (arrowheads).

Preoperative planning was based on the patient’s clinical manifestations and radiologic findings, including MRI and CT, to identify the target level and determine the extent of neural decompression (Figure 4). MRI was used to assess the level, severity, and distribution of neural compression, whereas CT provided a detailed assessment of the osseous anatomy, including the dimensions of the lamina, facet morphology, and the anticipated bony resection required for adequate decompression. These findings were integrated to determine the decompression strategy while minimizing the risk of unnecessary bone removal or neural injury.

#### 2.2.2. Surgeon-Directed Local Anesthesia with Full-Endoscopic Interlaminar Access

All procedures were performed under surgeon-directed local anesthesia designed to provide effective analgesia while preserving spontaneous respiration and continuous neurological communication. The anesthetic strategy incorporated stepwise local anesthesia, conscious sedation, MAC, and endoscopically guided epidural anesthesia [22,23,24,25]. Routine laboratory testing and clinical assessment were performed before surgery to assess hepatic and renal function and relevant medical conditions. Local anesthetic doses were reduced as needed based on body size, organ function, or overall clinical status. Patients were placed in a prone position on a radiolucent spinal table. Conscious sedation was initiated with intramuscular midazolam (0.05 mg/kg) and intravenous fentanyl (0.8 μg/kg), with additional doses titrated based on individual comfort and procedural requirements. Verbal responsiveness was maintained throughout the procedure to enable continuous neurological feedback. MAC was maintained by an anesthesiologist through continuous monitoring of blood pressure, heart rate, oxygen saturation, respiratory status, electrocardiography, level of consciousness, and neurological responsiveness while adjusting sedation to maintain spontaneous ventilation and patient communication. Local anesthetic serum concentrations were not routinely measured. Therefore, safety surveillance relied on dose adjustment and continuous clinical monitoring for neurological and cardiovascular signs of local anesthetic systemic toxicity.

A mixture containing up to 15 mL of 2% lidocaine (300 mg) and 15 mL of 0.75% ropivacaine (112.5 mg) was prepared for stepwise infiltration. This represented the maximum available amount rather than an amount that had to be administered in its entirety. Before endoscopic access, the smallest volume providing adequate analgesia was sequentially infiltrated into the skin, subcutaneous tissue, paraspinal muscles, periosteum, lamina, and facet capsule (Figure 1A), with the cumulative dose adjusted based on the patient’s body size, organ function, and clinical condition. When needed during docking or bony decompression, supplemental infiltration was taken from this prepared mixture rather than added beyond it. A 1 cm skin incision was made under fluoroscopic guidance, and sequential dilators were inserted to establish the interlaminar working corridor. The working sheath was docked at the ipsilateral lamina-facet junction, and a working channel endoscope (joimax GmbH, Karlsruhe, Germany) was introduced. Soft tissues overlying the lamina, facet joint, and ligamentum flavum were removed using endoscopic forceps and limited bipolar radiofrequency (joimax GmbH, Karlsruhe, Germany) under continuous saline irrigation maintained at approximately 30–40 mmHg. Subsequently, a limited laminotomy was performed using a high-speed endoscopic burr system (Nakanishi Inc., Kanuma, Tochigi, Japan) to expose the ligamentum flavum [7,8,9,10,11,12].

After limited laminotomy, the cranial margin of the ligamentum flavum was exposed, and a small midline epidural window was created. Adequate working space was confirmed under direct endoscopic visualization despite severe preoperative stenosis. The dura was identified, and its integrity was confirmed before needle advancement. A gently curved-tip spinal needle was directed away from the dura into the epidural space under continuous endoscopic visualization. Approximately 10 mL of 0.75% ropivacaine (75 mg) was administered slowly and incrementally without force, and the endoscopic field, neurological responsiveness, and hemodynamic status were continuously monitored. The procedure was paused for approximately 2 min to allow epidural diffusion before neural decompression (Figure 1B). This sequence was intended to avoid injecting into the maximally compressed canal before establishing a visually confirmed epidural corridor.

#### 2.2.3. FE-ULBD

Following endoscopically guided epidural anesthesia, ipsilateral laminotomy was continued using a high-speed burr and Kerrison punches (Figure 2A). The cranial and caudal laminae were progressively undercut to expose the ligamentum flavum. Ipsilateral lateral recess decompression was performed first, followed by contralateral decompression through an over-the-top approach beneath the base of the spinous process [24]. The contralateral lamina and medial facet were progressively undercut until adequate decompression of the contralateral lateral recess and traversing nerve root was achieved (Figure 2B). Throughout the bony decompression, the ligamentum flavum was intentionally preserved as a protective barrier over the neural structures (Figure 3A,B). After adequate osseous decompression had been achieved, the hypertrophic ligamentum flavum was carefully detached and removed piecemeal using endoscopic punches and forceps (Figure 2C and Figure 3C). Continuous saline irrigation maintained a clear operative field, whereas a bipolar radiofrequency probe was used for soft tissue ablation and hemostasis. Decompression was considered complete when the dural sac expanded freely with restoration of dural pulsation, both traversing nerve roots demonstrated unrestricted mobility, and bilateral neural decompression was confirmed under endoscopic visualization (Figure 2D and Figure 3D).

#### 2.2.4. Postoperative Management

Patients were encouraged to ambulate as soon as their general condition permitted after surgery. Neurological status and procedure-related complications were assessed immediately after surgery, and most patients were discharged within 24 h without complications. Postoperative MRI or CT was selectively obtained when clinically indicated, including persistent or recurrent symptoms, unexpected neurological findings, or at the patient’s request (Figure 4C). Patients were subsequently followed in the outpatient clinic to evaluate pain relief, functional recovery, and procedure-related complications.

### 2.3. Clinical Outcome Evaluation

Clinical outcomes were assessed using the visual analog scale (VAS) for leg pain and the Oswestry Disability Index (ODI) [26]. Outcome measures were recorded preoperatively and at 6 weeks, 6 months, and 12 months postoperatively. The primary endpoint of the study was the clinical outcome at 12 months. Overall patient satisfaction at the final follow-up was evaluated using the modified MacNab criteria (excellent, good, fair, or poor) [27], with favorable outcomes defined as excellent or good.

Perioperative variables, including operative time, length of hospital stay, and surgical complications, were also evaluated. Complications of interest included dural tears, epidural hematoma, surgical site infection, transient postoperative dysesthesia, neurological deterioration, and index-level revision surgery during the follow-up period.

For the clinical context, demographic characteristics, perioperative variables, complications, and overall clinical outcomes were also compared with those of younger patients who underwent single-level FE-ULBD during the same study period.

### 2.4. Comparison with Representative Published Studies

To provide external clinical context for the present findings, representative studies reporting clinical outcomes of lumbar decompression in older patients were additionally reviewed. Studies were selected if they evaluated older patients (generally aged 75 years or older or predominantly elderly cohorts) undergoing surgical decompression for lumbar spinal stenosis and reported sufficient demographic, perioperative, and clinical outcome data for comparison. The selected studies included conventional open decompression, microscopic decompression, minimally invasive decompression, ULBD, and endoscopic decompression techniques. Key patient characteristics, perioperative variables, clinical outcomes, complications, reoperation rates, and mortality were extracted for comparison with the present series.

### 2.5. Statistical Analysis

Statistical analyses were performed using Statistical Package for Social Sciences (version 22.0; IBM Corp., Armonk, NY, USA). Continuous variables were summarized as mean ± standard deviation, and categorical variables were summarized as frequencies and percentages. VAS and ODI score changes over time were evaluated using repeated-measures analysis of variance. Between-group comparisons were performed using independent-samples *t*-tests for continuous variables, Fisher’s exact tests for binary categorical variables, and Fisher–Freeman–Halton exact tests for categorical variables with more than two levels. All statistical tests were two-sided, and a *p*-value < 0.05 was considered statistically significant.

## 3. Results

### 3.1. Baseline Characteristics and Perioperative Findings

A total of 24 patients aged ≥75 years met the eligibility criteria and completed the 12-month follow-up. Of the 24 patients, 13 were males, and 11 were females, with a mean age of 80.46 ± 3.79 years (range, 75–85 years) and a mean body mass index (BMI) of 25.44 ± 3.07 kg/m^2^. L4–L5 was the most commonly treated level (*n* = 10), followed by L3–L4 (*n* = 8), L5–S1 (*n* = 5), and L2–L3 (*n* = 1). The mean operative time was 68.58 ± 16.54 min, and the mean length of hospital stay was 1.67 ± 0.87 days. The younger comparison group was included exclusively to provide a descriptive age-related context. No statistically significant differences in sex distribution, BMI, operative level, operative time, length of hospital stay, specific complication rates, reoperation rate, or overall clinical outcomes were observed between the two groups (Table 1).

In the older cohort, one patient (4.2%) was classified as ASA physical status I, 14 (58.3%) as ASA II, and 9 (37.5%) as ASA III. No patient was classified as ASA IV. The median age-unadjusted CCI was 1 (interquartile range, 0–1), and the median age-adjusted CCI was 4 (interquartile range, 4–5). Hypertension (75.0%) and diabetes mellitus (50.0%) were the most prevalent comorbidities, followed by coronary artery disease (16.7%), cerebrovascular disease (8.3%), and chronic pulmonary disease (8.3%). Table 2 shows the detailed comorbidity and anesthetic risk profile of the older cohort.

### 3.2. Clinical Improvement During Follow-Up

**Figure 5:**
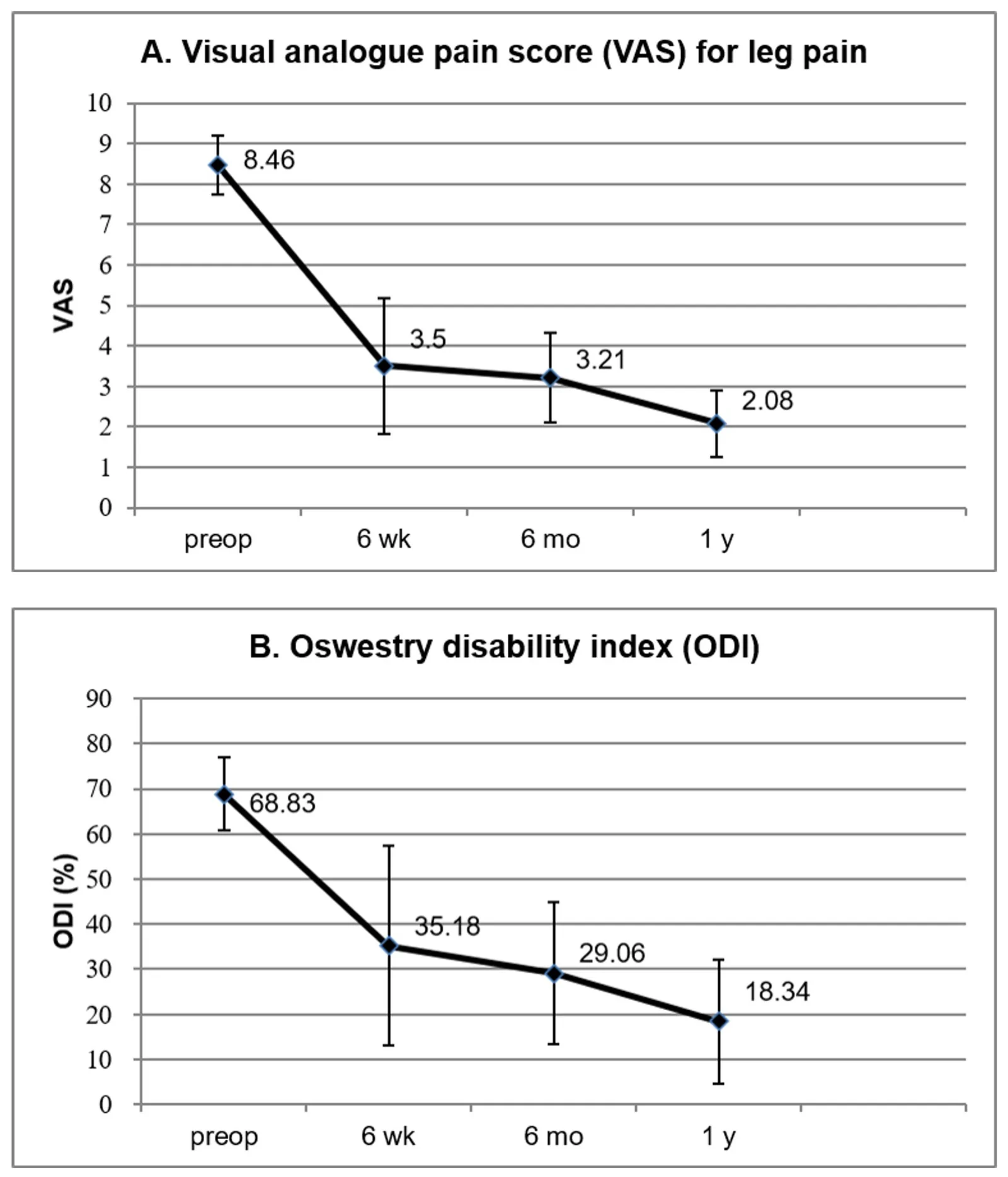
Changes in clinical outcomes following FE-ULBD in patients aged 75 years or older. (**A**) Mean VAS scores for leg pain obtained preoperatively and at 6 weeks, 6 months, and 12 months after surgery. (**B**) Mean ODI scores measured at the corresponding follow-up intervals.

Postoperative pain and function significantly improved and remained stable throughout follow-up. The mean VAS score for leg pain decreased from 8.46 ± 0.72 preoperatively to 3.50 ± 1.69, 3.21 ± 1.10, and 2.08 ± 0.83 at 6 weeks, 6 months, and 12 months, respectively (*p* < 0.001). Similarly, the mean ODI score improved from 68.83% ± 8.07% preoperatively to 35.18% ± 22.08%, 29.06% ± 15.75%, and 18.34% ± 13.74% at 6 weeks, 6 months, and the final follow-up (*p* < 0.001). The temporal changes in VAS and ODI scores are shown in Figure 5.

### 3.3. Patient Satisfaction and Safety Outcomes

**Figure 6:**
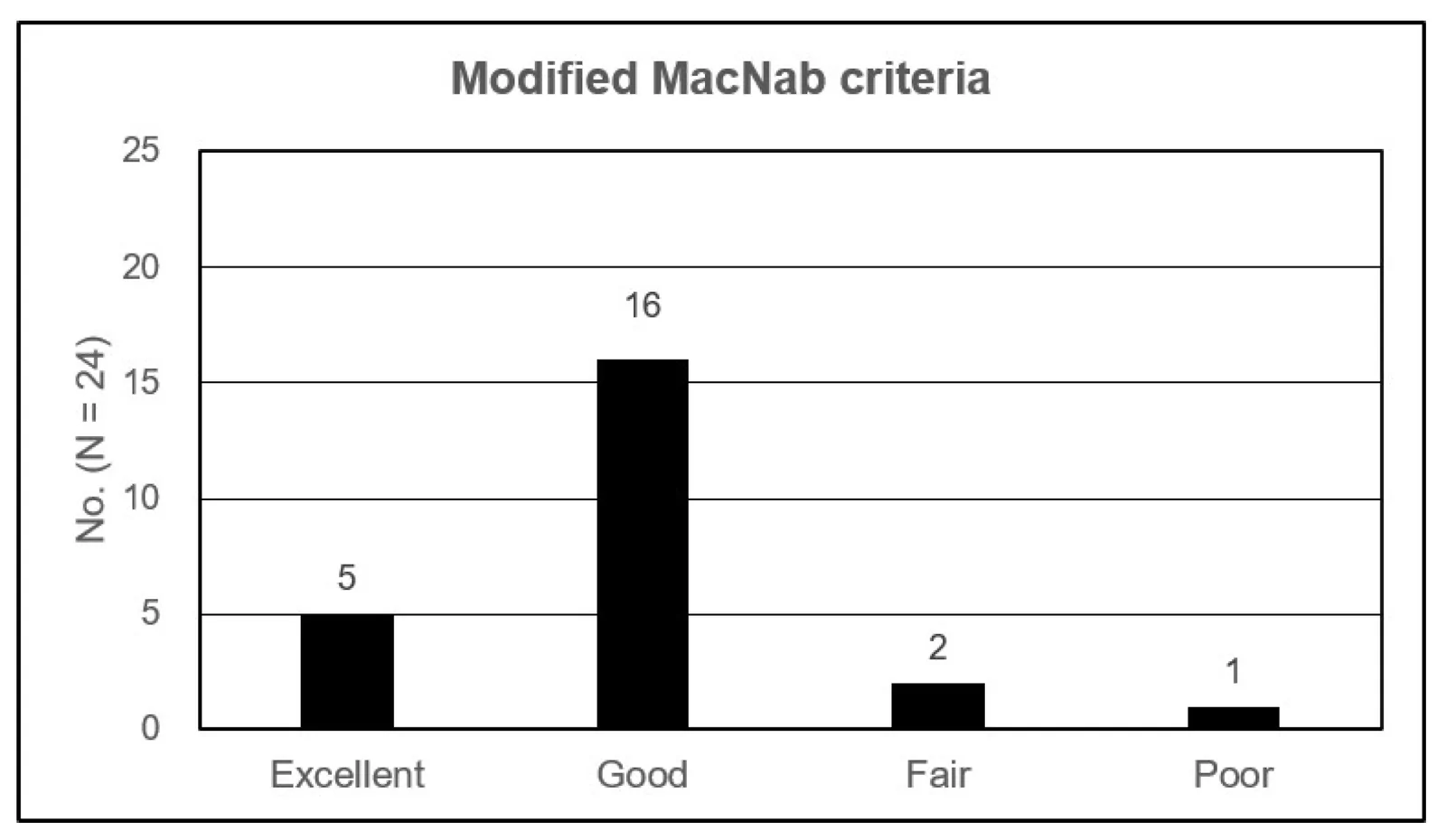
Distribution of global clinical outcomes according to the modified MacNab criteria at the 12-month follow-up. Outcomes were graded as excellent in 5 patients (20.8%), good in 16 (66.7%), fair in 2 (8.3%), and poor in 1 (4.2%). Favorable outcomes (excellent or good) were achieved in 21 of 24 patients (87.5%).

At 12 months, the modified MacNab outcomes were excellent in 5 patients (20.8%), good in 16 (66.7%), fair in 2 (8.3%), and poor in 1 (4.2%). Overall, 87.5% of patients (*n* = 21/24) achieved favorable outcomes (excellent or good) (Figure 6). No major medical, anesthetic, or surgical complications, including infections, epidural hematoma, cardiopulmonary events, permanent neurological decline, and revision surgery, occurred during the study. Procedure-related complications included transient postoperative dysesthesia in 4 patients (16.7%) and an incidental dural tear in 1 patient (4.2%). The dural tear was successfully managed intraoperatively without conversion to open surgery, and all cases of postoperative dysesthesia resolved with conservative treatment during follow-up. The two fair and one poor MacNab outcomes were clinically suspected to reflect residual or recurrent stenosis, although this explanation was not confirmed by systematic postoperative imaging. No case required revision surgery, and no direct causal relationship could be established retrospectively between these outcome categories and transient dysesthesia or the dural tear.

## 4. Discussion

### 4.1. Clinical Effectiveness of FE-ULBD Under Surgeon-Directed Local Anesthesia

This preliminary single-center retrospective cohort study of older patients with symptomatic lumbar spinal stenosis showed that FE-ULBD performed under surgeon-directed local anesthesia was associated with significant improvements in pain and function. The VAS and ODI scores improved during the follow-up period, and these changes persisted at 12 months. Additionally, 87.5% of patients had excellent or good MacNab outcomes. Lumbar decompression remains an established surgical treatment for persistent symptoms after failed conservative management [1,2]. However, concerns about perioperative morbidity often influence treatment decisions in older adults. The observed improvements exceeded the published thresholds for clinically meaningful improvement [28,29]. However, the absence of an older-patient control group precludes attributing these outcomes specifically to the anesthetic protocol.

These findings are consistent with those of previous studies on endoscopic decompression in older adults [13,14,15,16,17,18] and support further evaluation of minimally invasive decompression in carefully selected older patients. The perioperative outcomes in the descriptive younger cohort were not significantly different from those in the older cohort. However, this comparison addresses outcomes across age groups undergoing the same procedure and anesthetic protocol. It does not evaluate whether local anesthesia is more advantageous than general anesthesia in older patients. Accordingly, the younger cohort provides clinical context regarding age, not causal evidence for the benefit of the anesthetic strategy.

### 4.2. Comparison with Representative Studies of Lumbar Decompression in Older Patients

The results were compared with representative studies evaluating lumbar decompression surgery in older patients to better interpret the clinical significance of the present findings (Table 3) [3,30,31,32]. These studies encompass a broad spectrum of surgical strategies, including conventional open laminectomy, microscopic decompression, minimally invasive decompression, and full-endoscopic techniques. Although caution is warranted when interpreting direct comparisons due to differences in study design, patient selection, follow-up duration, and outcome assessment, several clinically meaningful observations can be drawn.

Lumbar decompression generally improves pain, function, and patient satisfaction in older patients. However, differences in anesthesia, procedure, case mix, follow-up, and complication definitions preclude direct comparisons between studies. In this study, the leg pain VAS score decreased from 8.46 to 2.08, the ODI score decreased from 68.83% to 18.34%, and 87.5% of patients had an excellent or good MacNab outcome. The mean hospital stay was 1.67 days, and no major cardiopulmonary or anesthesia-related complications were observed. These findings should be interpreted as descriptive feasibility observations rather than evidence of superiority over conventional decompression or general anesthesia.

The combination of FE-ULBD and a surgeon-directed local anesthesia protocol incorporating conscious sedation, MAC, and endoscopically guided epidural anesthesia is a distinctive feature of this study. This strategy was intended to limit the surgical and anesthetic burden while preserving patient communication. However, this study could not establish superiority or quantify any anesthesia-specific advantage because it did not include a comparator group of older patients receiving general anesthesia. The literature comparison provides contextual benchmarking only.

### 4.3. Clinical Advantages of Surgeon-Directed Local Anesthesia in Older Patients

Older patients with lumbar spinal stenosis often present with multiple medical comorbidities, reduced physiological reserve, and an increased risk of delayed recovery following conventional surgery [3,4,5]. Although minimally invasive decompression reduces surgical trauma, the choice of anesthetic strategy also may play an important role in perioperative recovery, particularly in medically vulnerable older adults [33,34]. Therefore, an anesthetic strategy that minimizes physiological stress while maintaining procedural safety is of particular clinical value in this population.

The surgeon-directed local anesthesia protocol used in the present study was designed to provide effective analgesia while preserving spontaneous respiration, verbal communication, and continuous neurological feedback throughout the procedure. Rather than relying on a single anesthetic technique, analgesia was progressively adjusted according to each stage of surgery through stepwise local infiltration, conscious sedation, MAC, and endoscopically guided epidural anesthesia [22,23,24,25]. This flexible approach allowed the operating surgeon to supplement local analgesia when discomfort was anticipated during docking, laminotomy, or neural decompression, thereby maintaining patient comfort without unnecessarily increasing systemic sedation.

From a practical perspective, this strategy integrates analgesic supplementation into the surgical workflow while preserving spontaneous respiration and neurological communication. No major anesthesia-related complication was observed in this series, and early mobilization and discharge were generally achieved. These findings support procedural feasibility in selected patients but do not establish comparative safety or effectiveness with general or neuraxial anesthesia.

These advantages complement the inherent benefits of the full-endoscopic unilateral approach, which minimizes soft tissue injury while preserving posterior stabilizing structures [6,35]. In carefully selected patients without radiographic instability, this strategy may also avoid unnecessary fusion procedures, which have not consistently demonstrated superior outcomes over decompression alone but are associated with greater surgical invasiveness [36]. Recent reports have likewise supported the feasibility of endoscopic decompression performed under local anesthesia in medically vulnerable elderly patients [37,38].

In this study, transient postoperative dysesthesia occurred in 4 patients (16.7%) and resolved with conservative treatment. Although the underlying mechanism could not be determined, it was likely multifactorial. Potential contributing mechanisms include mechanical irritation during ipsilateral or over-the-top contralateral decompression, traversing root manipulation, radiofrequency thermal effects, irrigation-related pressure, and postoperative inflammatory neuritis. In our technique, radiofrequency use was limited, and irrigation pressure was maintained at approximately 30–40 mmHg, making major thermal or pressure injury less likely but not excluding their contribution. Local anesthetic rebound hyperalgesia could not be confirmed or excluded because no systematic mechanistic assessment was performed. The incidence of 16.7% warrants explicit reporting and further evaluation in larger prospective studies.

### 4.4. Technical Considerations for FE-ULBD Under Surgeon-Directed Local Anesthesia

Several technical aspects contribute to the successful implementation of FE-ULBD under surgeon-directed local anesthesia. Unlike conventional anesthetic protocols in which anesthetic management is largely predetermined before surgery, our protocol allows local analgesia to be progressively supplemented according to procedural requirements. This stepwise strategy maintains patient comfort throughout docking, laminotomy, and neural decompression while preserving spontaneous communication and neurological responsiveness.

A distinctive component of our technique is endoscopically guided epidural anesthesia after limited laminotomy. The injection was performed only after a midline epidural corridor was created and the dura and surrounding space were directly visualized. Slow incremental injection without force and continuous endoscopic, neurological, and hemodynamic observation were used to reduce the risks of inadvertent dural injury and abrupt pressure elevation. Although its independent effect was not evaluated, this step might facilitate tolerance during ligamentum flavum removal and bilateral neural decompression [23,38]. However, this observation remains hypothesis-generating.

Another important technical consideration is achieving adequate bilateral neural decompression through a unilateral working corridor while preserving stabilizing posterior structures. Progressive undercutting of the laminae and preservation of the ligamentum flavum during bony decompression minimize unnecessary tissue disruption while facilitating safe contralateral decompression [6,12,13,14]. Rather than emphasizing the extent of bone removal, adequate decompression should be confirmed by restoration of dural pulsation, full expansion of the dural sac, and unrestricted mobility of both traversing nerve roots under endoscopic visualization.

Finally, the successful application of this technique requires proficiency in advanced endoscopic decompression and surgeon-directed local anesthesia. Appropriate patient selection, meticulous preoperative planning, and progressive acquisition of technical experience remain essential for achieving safe, reproducible, and durable clinical outcomes [15,16].

### 4.5. Limitations

This study has some limitations. First, this was a single-center retrospective study with a small sample size, which may introduce selection bias and provide insufficient power to detect rare anesthesia-related or surgical adverse events. Second, all procedures were performed by a highly experienced endoscopic spine surgeon, which may limit the generalizability of the findings. Third, the 12-month follow-up did not establish long-term durability. Fourth, although the ASA physical status and CCI were reconstructed from the available preoperative records, this retrospective assessment cannot capture all frailty, cardiopulmonary reserve, and anesthesia-specific risk dimensions. Furthermore, 62.5% of patients were ASA I or II, and none were ASA IV. Therefore, the cohort should not be characterized as uniformly high-risk or unsuitable for general anesthesia. Fifth, the younger comparator cohort underwent the same local-anesthesia protocol and therefore informs age-related context only; without an age-matched older cohort treated under general anesthesia, neither comparative anesthetic benefit nor the independent contribution of endoscopically guided epidural anesthesia can be determined. Sixth, local anesthetic serum concentrations were not measured, and pharmacokinetic safety margins could not be defined. Finally, the two fair and one poor MacNab outcomes were clinically suspected to reflect residual or recurrent stenosis, although this interpretation was not confirmed by systematic postoperative imaging. The retrospective data also did not permit causal linkage of these outcomes to specific complications. Therefore, further larger prospective multicenter studies with prospectively standardized frailty and anesthetic risk measures, predefined adverse-event surveillance, patient-level outcome characterization, and an appropriate older-patient anesthesia comparator are needed.

## 5. Conclusions

FE-ULBD under surgeon-directed local anesthesia was feasible and associated with improved pain and function at 12 months in carefully selected patients aged ≥75 years. These findings provide preliminary evidence of feasibility but do not establish superiority over general anesthesia or other decompression strategies. Further prospective comparative studies are needed to evaluate safety, effectiveness, and generalizability.

## Figures and Tables

**Table 1:** Comparison of demographic and perioperative characteristics between older and younger patients undergoing single-level FE-ULBD.

	Older ≥ 75 Years (*n* = 24)	Younger (*n* = 95)	*p*-Value
Age (years)	80.46 ± 3.79 (75–85)	59.71 ± 10.49 (36–74)	<0.001
Male:female	13:11	60:35	0.484
Body mass index	25.44 ± 3.07	25.32 ± 3.42	0.876
Operative level			0.162
L1–2	0	1	
L2–3	1	0	
L3–4	8	17	
L4–5	10	50	
L5–S1	5	27	
Operative time (min)	68.58 ± 16.54	68.31 ± 17.13	0.945
Hospital stay (days)	1.67 ± 0.87	1.36 ± 0.87	0.122
MacNab criteria			0.280
Excellent	5 (20.8%)	26 (27.4%)	
Good	16 (66.7%)	65 (68.4%)	
Fair	2 (8.3%)	3 (3.2%)	
Poor	1 (4.2%)	1 (1.1%)	
Reoperation (%)	0	2	1.000
Complication (%)			
Infection	0	0	1.000
Hematoma	0	1	1.000
Dural tear	1 (intraoperative repair)	5 (intraoperative repair)	1.000
Dysesthesia	4 (transient)	13 (transient)	0.746

**Table 2:** Preoperative comorbidity and anesthetic risk profile of the older cohort (*n* = 24).

Variable	Older Cohort (*n* = 24)
ASA physical status	
I	1 (4.2%)
II	14 (58.3%)
III	9 (37.5%)
IV	0 (0%)
Age-unadjusted CCI, median (IQR)	1 (0–1)
Age-adjusted CCI, median (IQR)	4 (4–5)
Hypertension	18 (75.0%)
Diabetes mellitus	12 (50.0%)
Coronary artery disease	4 (16.7%)
Cerebrovascular disease	2 (8.3%)
Chronic pulmonary disease	2 (8.3%)
Renal disease requiring dialysis	1 (4.2%)
Parkinson’s disease	2 (8.3%)
Dementia	0 (0%)
Rheumatologic disease	0 (0%)
Depression	1 (4.2%)
Benign prostatic hyperplasia	2 (8.3%)
Dyslipidemia	1 (4.2%)

Data are presented as numbers (%) unless otherwise indicated. ASA, American Society of Anesthesiologists; CCI, Charlson Comorbidity Index; IQR, interquartile range.

**Table 3:** Comparison of representative studies of lumbar decompression surgery in older patients.

Variable	Rosen et al. (2007) [31]	Shabat et al. (2008) [30]	Jakola et al. (2010) [3]	Gerhardt et al. (2018) [32]	Present Study
Study characteristics					
Age criterion	≥75 years	≥80 years	≥70 years	≥80 years	≥75 years
Patients analyzed	57 treated; 50 with outcomes	39 treated; 25 at follow-up	101	244	24
Study design	Retrospective cohort	Prospective cohort	Prospective cohort	Retrospective cohort	Retrospective cohort
Procedural and perioperative characteristics					
Procedure	Microendoscopic decompression, discectomy, or foraminotomy; no fusion	Complete laminectomy ± discectomy; no fusion	Conventional laminectomy without fusion	Open microsurgical decompression ± tubular approach; no stabilization	Single-level FE-ULBD
Anesthesia	NR	General anesthesia	NR	NR	Surgeon-directed local anesthesia with conscious sedation and MAC
Follow-up	Median 7 months (mean 10 months)	Mean 36.8 months	12 months	Perioperative/90-day outcomes	12 months
Length of stay	Median 29 h (mean 53 h)	Mean 3.6 days	Median 6 days	Mean 9.0 ± 7.4 days	Mean 1.67 ± 0.87 days
Clinical outcomes and safety					
Representative Clinical Outcomes	Back VAS 5.7 → 2.2; leg VAS 5.7 → 2.3; ODI 48 → 27	VAS 8.84 → 3.60; Barthel 62.8 → 77.0; 76% satisfied	ODI 44.2 → 27.9; leg VAS 60.2 → 33.6; 86.9% reported benefit	91% symptom improvement at discharge; 63.2% neurologic improvement	Leg VAS 8.46 → 2.08; ODI 68.83 → 18.34; MacNab excellent/good 87.5%
Reported Complications	38.6% (22/57); chiefly urinary retention and transient delirium	52%; approximately half minor	18.0% overall; 6.0% major	22.5% intraoperative; 7.0% postoperative medical	4.2% dural tear; 16.7% transient dysesthesia
Reoperation	1.8% (1/57)	4.0% (1/25)	2.0% within 12 mo	4.1% during index admission; 11.5% after discharge	0%
Serious Adverse Events	No major complication or perioperative death	No operative or perioperative death; 1 CVA (4%)	1 perioperative death (1.0%)	Mortality 0.8%; severe medical complications in 14 events	No major cardiopulmonary event, permanent neurologic deterioration, or mortality
Distinctive Feature	Early elderly MIS decompression series	Long-term octogenarian open decompression cohort	Prospective conventional laminectomy cohort	Largest octogenarian microsurgical decompression cohort	FE-ULBD with surgeon-directed local anesthesia and endoscopically guided epidural anesthesia

CVA, cerebrovascular accident; FE-ULBD, full-endoscopic unilateral laminotomy for bilateral decompression; MAC, monitored anesthesia care; MIS, minimally invasive surgery; NR, not reported; ODI, Oswestry Disability Index; VAS, visual analog scale. VAS values were reported on a 0–10 scale except for Jakola et al., who used a 0–100 scale.

## Data Availability

The data presented in this study are available from the corresponding author upon reasonable request. The data are not publicly available due to patient privacy and institutional ethical restrictions.

## Abbreviations

The following abbreviations are used in this manuscript:

ASA	American Society of Anesthesiologists
BMI	Body mass index
CCI	Charlson Comorbidity Index
CT	Computed tomography
FE-ULBD	Full-endoscopic unilateral laminotomy for bilateral decompression
IQR	Interquartile range
MAC	Monitored anesthesia care
MRI	Magnetic resonance imaging
ODI	Oswestry disability index
VAS	Visual analog scale
